# Generation of induced pluripotent stem cells from large domestic animals

**DOI:** 10.1186/s13287-020-01716-5

**Published:** 2020-06-25

**Authors:** Fabiana Fernandes Bressan, Vinícius Bassanezze, Laís Vicari de Figueiredo Pessôa, Chester Bittencourt Sacramento, Tathiane Maistro Malta, Simone Kashima, Paulo Fantinato Neto, Ricardo De Francisco Strefezzi, Naira Caroline Godoy Pieri, José Eduardo Krieger, Dimas Tadeu Covas, Flávio Vieira Meirelles

**Affiliations:** 1 Department of Veterinary Medicine, Faculty of Animal Sciences and Food Engineering, University of São Paulo, Pirassununga, Brazil; 2 Postgraduate Program in Anatomy of Domestic and Wild Animals, School of Veterinary Medicine and Animal Science, University of São Paulo, São Paulo, Brazil; 3 Center for Cell-Based Therapy, Regional Blood Center, School of Medicine of Ribeirão Preto, University of São Paulo, Ribeirão Preto, Brazil; 4 Heart Institute (INCOR), Faculty of Medicine, University of São Paulo, São Paulo, Brazil; 5 Present Address: Brigham and Women’s Hospital, Harvard Medical School, Boston, USA; 6 Present Address: Weill Cornell Medicine, Cornell University, Ithaca, USA; 7 School of Pharmaceutical Sciences of Ribeirão Preto, University of São Paulo, Ribeirão Preto, SP Brazil

**Keywords:** Bovine, Cellular reprogramming, Equine, Induced pluripotency, iPSCs, stem cells

## Abstract

**Background:**

Induced pluripotent stem cells (iPSCs) have enormous potential in developmental biology studies and in cellular therapies. Although extensively studied and characterized in human and murine models, iPSCs from animals other than mice lack reproducible results.

**Methods:**

Herein, we describe the generation of robust iPSCs from equine and bovine cells through lentiviral transduction of murine or human transcription factors Oct4, Sox2, Klf4, and c-Myc and from human and murine cells using similar protocols, even when different supplementations were used. The iPSCs were analyzed regarding morphology, gene and protein expression of pluripotency factors, alkaline phosphatase detection, and spontaneous and induced differentiation.

**Results:**

Although embryonic-derived stem cells are yet not well characterized in domestic animals, generation of iPS cells from these species is possible through similar protocols used for mouse or human cells, enabling the use of pluripotent cells from large animals for basic or applied purposes. Herein, we also infer that bovine iPS (biPSCs) exhibit similarity to mouse iPSCs (miPSCs), whereas equine iPSs (eiPSCs) to human (hiPSCs).

**Conclusions:**

The generation of reproducible protocols in different animal species will provide an informative tool for producing in vitro autologous pluripotent cells from domestic animals. These cells will create new opportunities in animal breeding through transgenic technology and will support a new era of translational medicine with large animal models.

## Background

Induced pluripotent stem cells (iPSCs) have enormous potential in cellular therapy, reprogramming, and early development approaches as they can differentiate into numerous autologous cell lineages, including the three germ layers. Since the major breakthrough of iPSC generation in 2006 [1], several studies have reproduced the induction of cellular pluripotency through overexpression of specific transcription factors. Although some reports describe iPSC generation in dogs, pigs, horses, cattle, and some other species as reviewed elsewhere [2], the most thoroughly characterized iPSC cells are undoubtedly from humans and mice [3–16].

Interestingly, iPSCs can be generated from species that do not have well-characterized embryonic-derived pluripotent stem cells (ES cells) yet. For example, in bovine and equine models, the lack of characterization of pluripotent features leads to their denomination as embryonic “stem cell-like” cells [17, 18], although recent advances have been reported recently in cattle [19]. The clinical use of iPSCs is still limited by several factors, including the low efficiency of reprogramming and the lack of studies assuring the safety of transplantation procedures, a consequence of unstable karyotypes and genomic alterations due to viral integration. iPSC production, therefore, must be improved to be reproducible and safe for its therapeutic use to become possible [20].

Though mice are still the most studied and used model for basic and applied research, it has considerable limitations, in special, in early development, and in overall physiology when compared to humans [21]. Rabbits are also considered adequate models for translational research [22, 23]; however, its in vitro cell culture and pluripotency acquisition still lack further robust characterization [24]. In this context, domestic animal species such as swine, small ruminants, and even companion animals such as dogs may provide an important contribution to regenerative and translational medicine. iPSC technology suggests that although species-specific differences are evident, the basic mechanisms of pluripotency acquisition may follow similar patterns in mammals, and iPSCs have been reported from these animals [25–27]. Specifically, large farm animals are important models not only for pre-clinical stem cell therapies due to their physiological and morphological similarity to humans [28, 29], but they also may greatly benefit from pluripotency in vitro to the generation of genetically superior or modified organisms for agricultural and biomedical applications, for example, for reproductive sciences [30].

A deeper understanding of the process of acquisition and maintenance of pluripotency and reprogramming in large domestic models will allow the optimization of several reproductive biotechnologies, the development of genetically engineered herds that may be useful as pre-clinical models for gene and cellular therapies, enhanced animal breeding programs and bioreactors [30]. This study tested whether the mechanisms used to induce pluripotency in human and mouse models can generate and maintain pluripotency in bovine and equine cells. Similarities and differences between the species in which ES cells were or were not reported yet are described and discussed.

## Methods

All procedures were performed in accordance with the Guide for the Care and Use of Laboratory Animals of the National Institutes of Health and The ARRIVE Guidelines, as well as with the rules issued by the National Council for Control of Animal Experimentation (CONCEA, Ministry of Science, Technology and Innovations and Communications, and in accordance with Law 11.794 of October 8, 2008, Decree 6899 of July 15, 2009). Protocols were then approved by the Ethics Committee on Animal Use of the School of Veterinary Medicine and Animal Science, University of São Paulo, Brazil (protocol number 2913/2013), and by the Ethics Committee on the Use of Animals of the Faculty of Animal Science and Food Engineering, University of São Paulo, Brazil (protocols number 3526250717 and 2192250918).

### Primary cell isolation and culture

Fibroblasts and adipose tissue-derived mesenchymal cells (AdMsc) were used in this study. Bovine (*Bos taurus* × *Bos indicus*) fetal fibroblasts (bFF) were isolated from a 50-day gestation fetus, murine (*Mus musculus*) fetal fibroblasts (mFF) were obtained from a 13-day gestation pool of fetuses, equine (*Equus caballus*) fibroblasts were derived from adult females (eAF), and human (*Homo sapiens*) fibroblasts used were acquired commercially (hAF, HDFa, Thermo Scientific). Bovine, human, and equine mesenchymal cells (bAdMSCs, hAdMSC, eAdMSCs, respectively) were derived from approximately 2 cm^3^ of adipose tissue, minced and incubated for 3 h at 38.5 °C in 0.040 g/mL collagenase IV (Sigma Aldrich). Fibroblasts and AdMsc were maintained in Iscove’s Modified Dulbecco’s Media (IMDM, Life Technologies) supplemented with 10% fetal bovine serum (Hyclone) and antibiotics (Life Technologies).

### Induction of pluripotency

A minimum of 3 independent replicates were performed for each cell lineage for pluripotency induction. All cell lineages used were under 10 cell passages.

Polycistronic lentiviral vectors (stem cell cassette (STEMCCA) [31]) containing human OCT4, SOX2, c-MYC, and KLF4 (hOSKM) or murine OSKM (mOSKM) were produced through 293FT lipofection (Lipofectamine 2000, Life Technologies) as described previously [32]; however, we used a ratio of 6:1:2 μg of OSKM; auxiliary vectors REV, TAT, and hgpm2; and packaging vector VSVG, respectively.

Transduction was performed overnight [32, 33], and 5 or 6 days after transduction, the cells were transferred to mitotically inactivated mouse embryonic fibroblasts (MEFs) and cultured for at least 14 days. iPSC medium consisted of DMEM/F12 KO (Life Technologies) supplemented with 20% KSR (Life Technologies), 1% glutamine (Life Technologies), 1:1000 β-mercaptoethanol (Life Technologies), 1% non-essential amino acids (Life Technologies), 10 ng/mL basic fibroblast growth factor (bFGF, Peprotech), antibiotics (pen/strep, Life Technologies), and, when specified, leukemia inhibitor factor (mouse or human -hLIF or hLIF, 1000 U/mL, Millipore, for mouse and human iPSCs, respectively) and 2i (two inhibitor - GSK3 inhibitor or iGSK3, 3.3 μM, Stemgent and MEK inhibitor or iMEK, 1 μM, Stemgent).

The first cell passage was performed manually in all experiments, and clonal lineages were then cultured in vitro. Human iPSCs (hiPSCs) did not respond well to enzymatic digestion with collagenase, trypsin, or dispase (data not shown). Murine, bovine, and equine (miPSCs, biPSCs, eiPSCs) iPSCs were dissociated from each other after a second passage with TrypLE Express (Life Technologies).

#### Induced pluripotent cell characterization

Cell cultures were visually assessed every 2 days for morphological changes. Alkaline phosphatase (AP) staining was performed with the Leukocyte Alkaline Phosphatase Kit (Sigma) following the manufacturer’s directions. At least 3 lines of biPSCs, eiPSCs, hiPSCs, and miPSCs were maintained for a minimum of 10 passages. At least one lineage of biPSCs was maintained for more than 50 passages, 1 lineage of eiPSCs and of hiPSCs for 30 passages, and 1 of miPSCs for more than 10 passages, and then cryopreserved.

Immunofluorescence of OCT4 protein was based on a protocol described in Oliveira et al. [34]. Briefly, iPSC cell cultures were fixed in 4% PFA for 20 min and maintained at 4 °C in PBS supplemented with 3% BSA and 0.5% Triton X-100 for a minimum of 12–16 h. Cell cultures were incubated in a blocking solution (PBS supplemented with 3% BSA and 0.2% Tween-20) for 1 h at room temperature. Cells were incubated with the primary antibody (OCT4 - rabbit anti OCT3/4, 1:50, Sigma) for 12–16 h at 4 °C, washed, and subsequently incubated with secondary antibodies (goat anti-rabbit 488, 1:100, Alexa Fluor, Invitrogen) for 2 h. Additionally, cells were incubated without primary antibodies for control to assess the immunofluorescence technique. Detection of NANOG was performed in biPSCs and miPSCs through immunofluorescence as described above (rabbit anti-NANOG, 1:100, Abcam 80892) and by RT-qPCR analysis in hiPSCs (Power SYBR Green, Life Technologies, F: 5′ CCAAAGGCAAACAACCCACTT 3′, R: 5′ CGGGACCTTGTCTTCCTTTTT 3′).

Embryoid bodies (EBs) were produced by seeding iPSC cells in dishes previously treated with 0.6% agarose and cultured in the absence of bFGF for 48–60 h. For in vitro induction of spontaneous differentiation, EBs were plated in dishes previously treated with 0.1% gelatin for a minimum of 6 days in DMEM/F12 KO supplemented with 20% FBS and antibiotics.

For in vivo differentiation assay, iPSC equine, bovine, and human as well as control cells (not reprogrammed) were injected subcutaneously in BALB/c nude female mice. One to four injections of approximately 1.5 × 10^6^ cells in 30% Matrigel (BD Biosciences) in PBS were injected per animal. Tumors, when present, were collected, fixed in PFA 4%, processed for histopathology, and stained with hematoxylin and eosin stain (H&E) for microscopic evaluation [35].

## Results

**Fig. 1:**
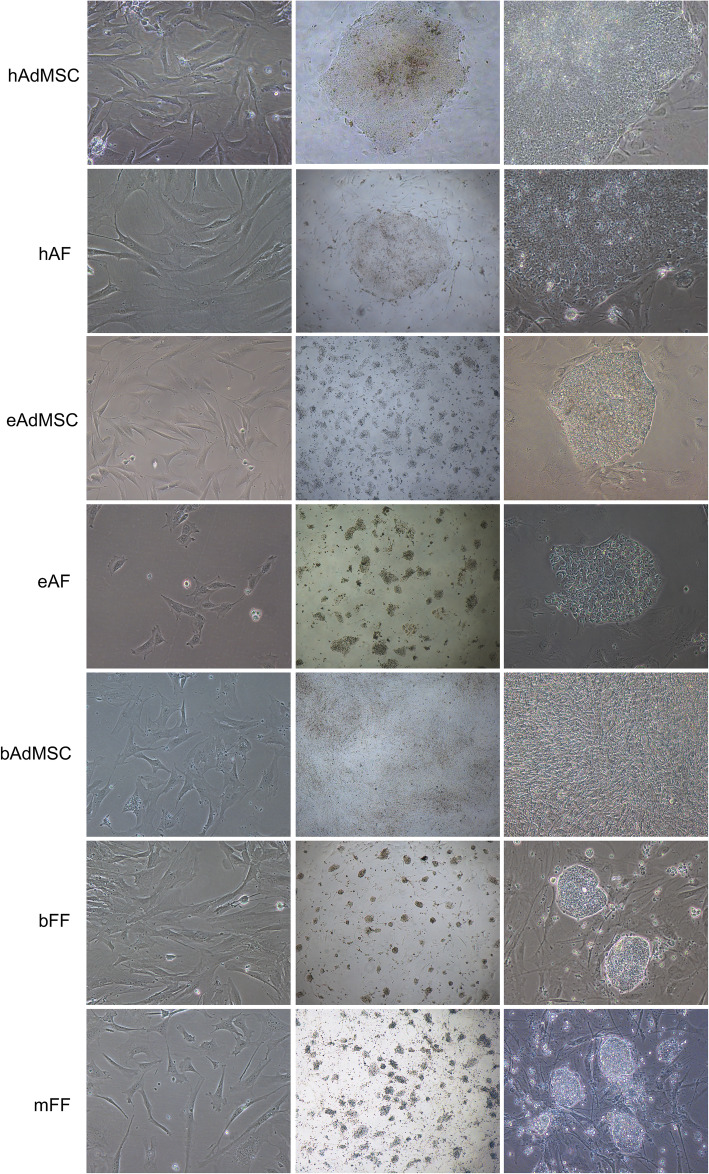
hAdMSC, hAF, eAdMSC, eAF, bAdMSC, bFF, and mFF throughout in vitro cellular reprogramming: before transduction (× 200) and iPS colonies after replating (× 40 and × 200)

Human cells showed the first signs of morphological differentiation into pluripotent cells approximately 10 days after transduction. Human adult fibroblasts (hAF) and human adipose-derived mesenchymal cells (hAdMSC) displayed initial colony formation approximately 5 days after transduction, and colonies were picked approximately 20 days after transduction (Fig. 1).

Enzymatic dissociation of hiPSCs did not allow culture viability after incubation with TrypLE Express or Dispase (BD Biosciences); therefore, hiPSCs were manually passaged throughout the experiments.

**Fig. 2:**
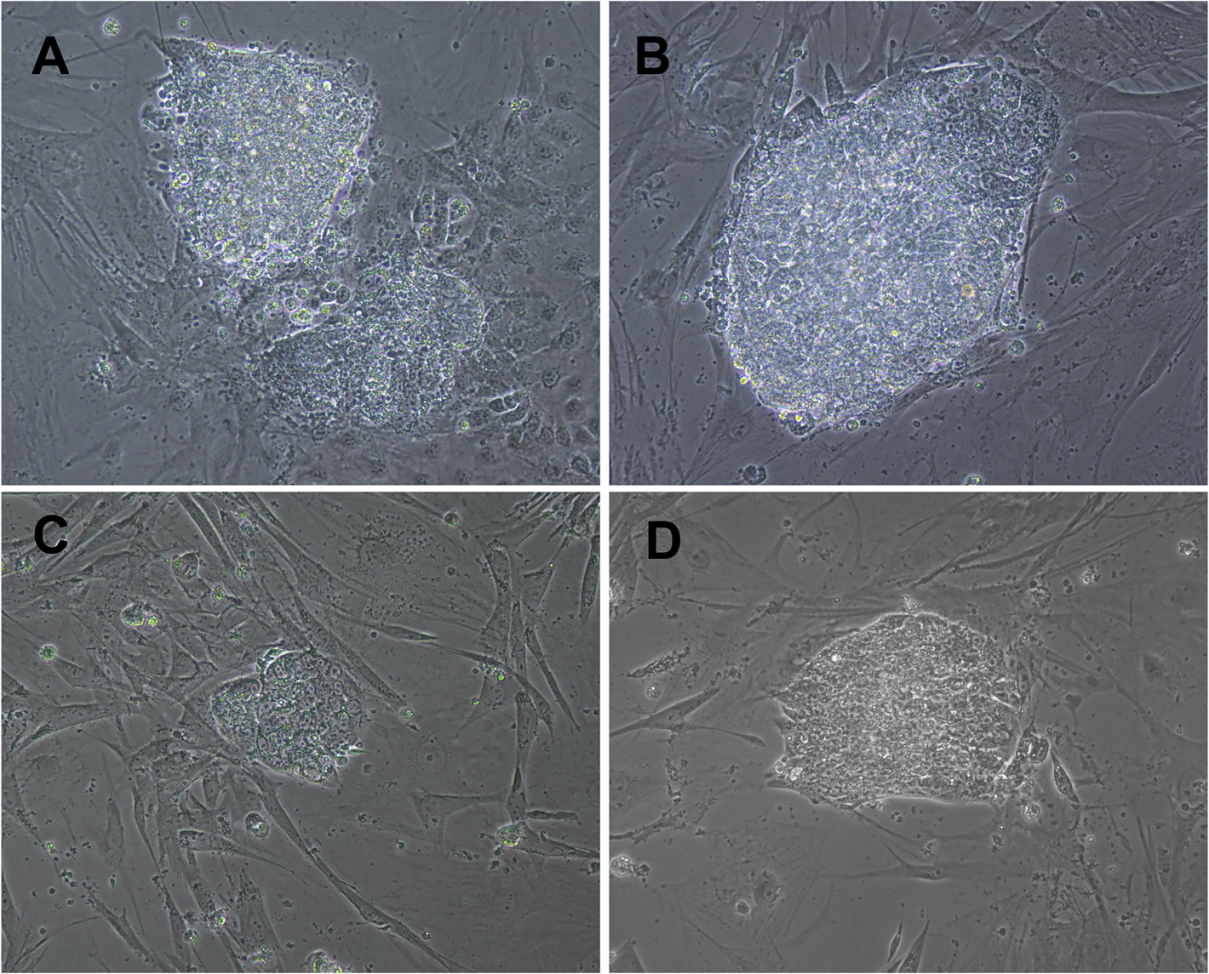
**a**, **b** Bovine in vitro reprogrammed cells (p0) with murine OSKM non-supplemented and supplemented with 2i+LIF, respectively. **c**, **d** Equine in vitro reprogrammed cells (p0) with human OSKM non-supplemented and supplemented with 2i+LIF, respectively (× 200)

Equine adult fibroblasts (eAF) and adipose tissue mesenchymal cells (eAdMSCs) were transduced with murine or human OSKM, which resulted in induced colonies approximately 3 days after transduction only with human OSKM (Fig. 1) that were independent of hLIF/2i supplementation (Fig. 2). Cells were replated approximately 15 days after transduction.

**Fig. 3:**
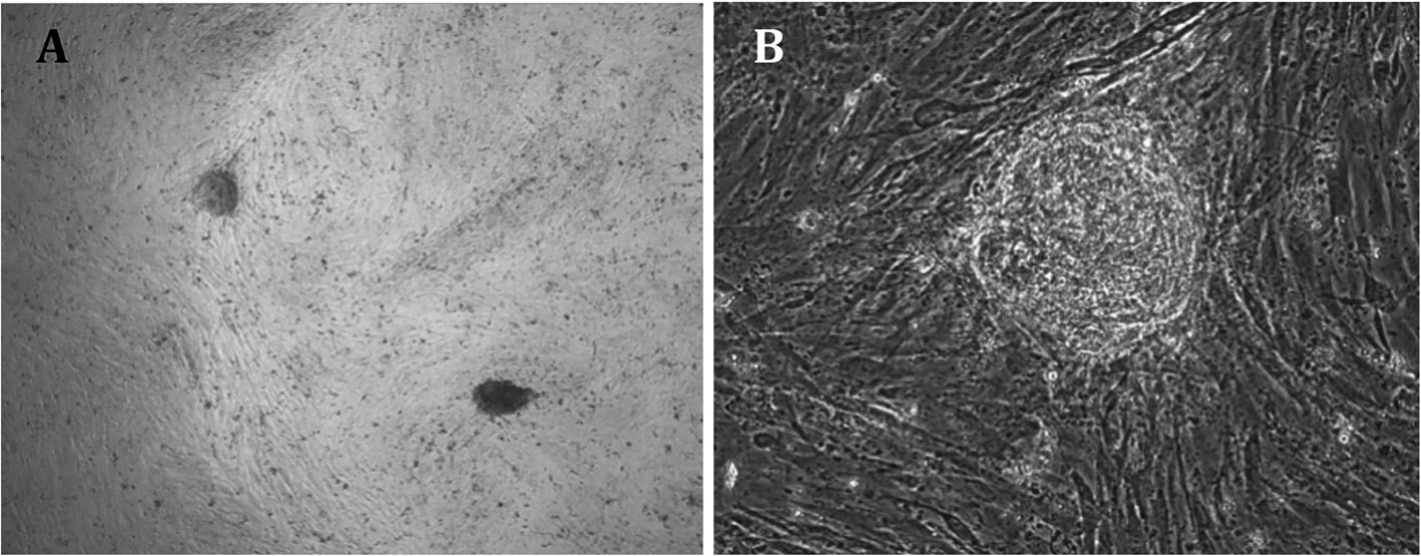
Non-replicative cell colonies of bFF transduced with hOSKM. **a** × 40. **b** × 200

Bovine fetal fibroblasts (bFF) and adipose tissue mesenchymal cells (bAdMSCs) were transduced with murine or human OKSM, and each group was cultured in iPSC medium supplemented with 2i+LIF or not. After approximately 10 days, bFF cells transduced with murine OSKM developed colonies morphologically similar to mouse iPSCs or ES cells. bFF cells transduced with human OSKM developed non-replicative cell colonies approximately 30 days post-transduction (Fig. 3). The bAdMSC lineage used in this study did not develop colonies after transduction with either hOSKM or mOSKM.

Murine fetal fibroblasts (mFF) were transduced with murine OSKM, and the first colonies developed approximately 3 days after transduction and were clonally replated after 15 days. Only colonies cultured with LIF maintained typical morphology after replating.

**Fig. 4:**
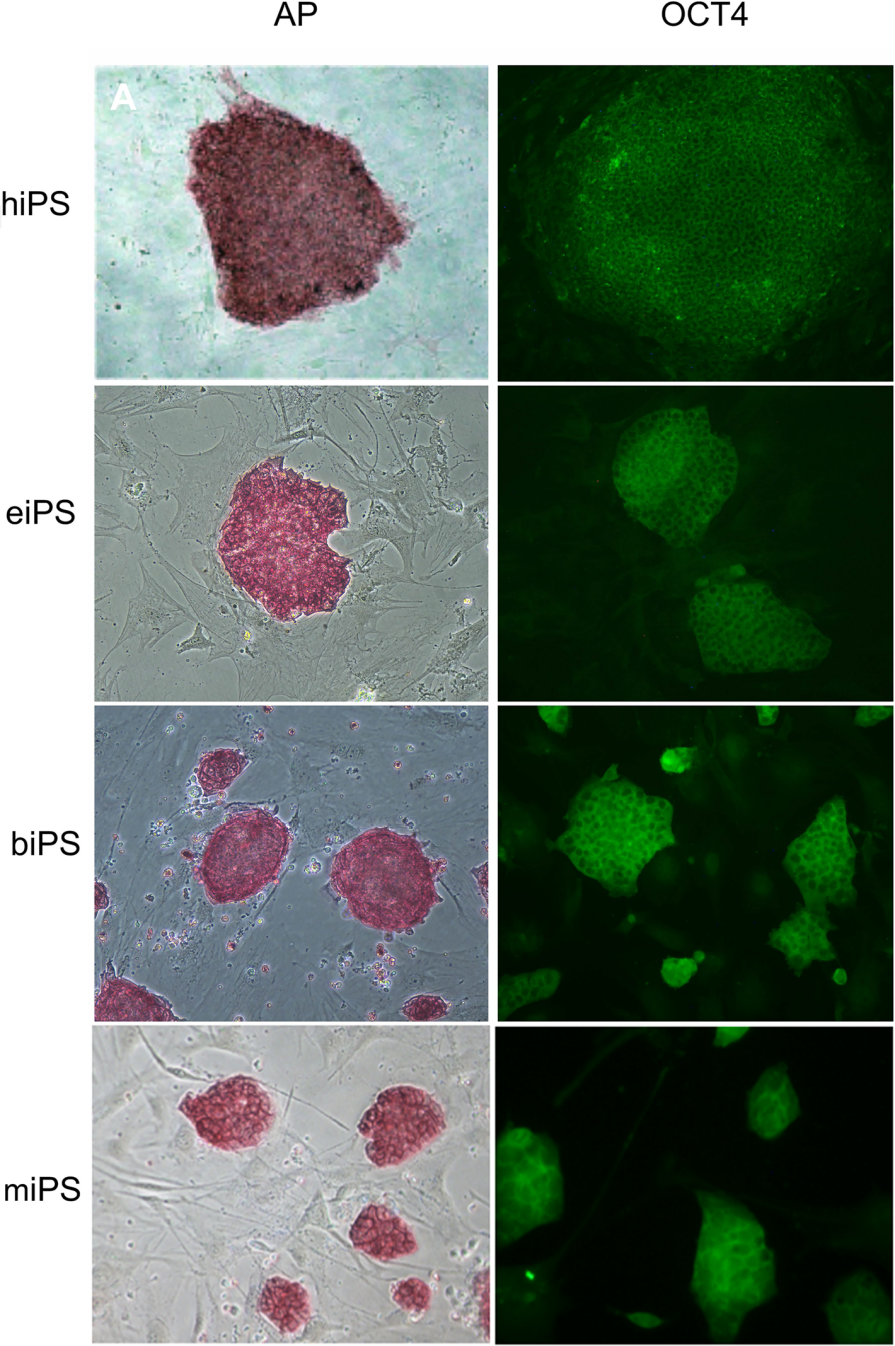
AP detection and immunofluorescence of OCT4 in iPS colonies derived from human, equine, bovine, and mouse cells. × 200

**Fig. 5:**
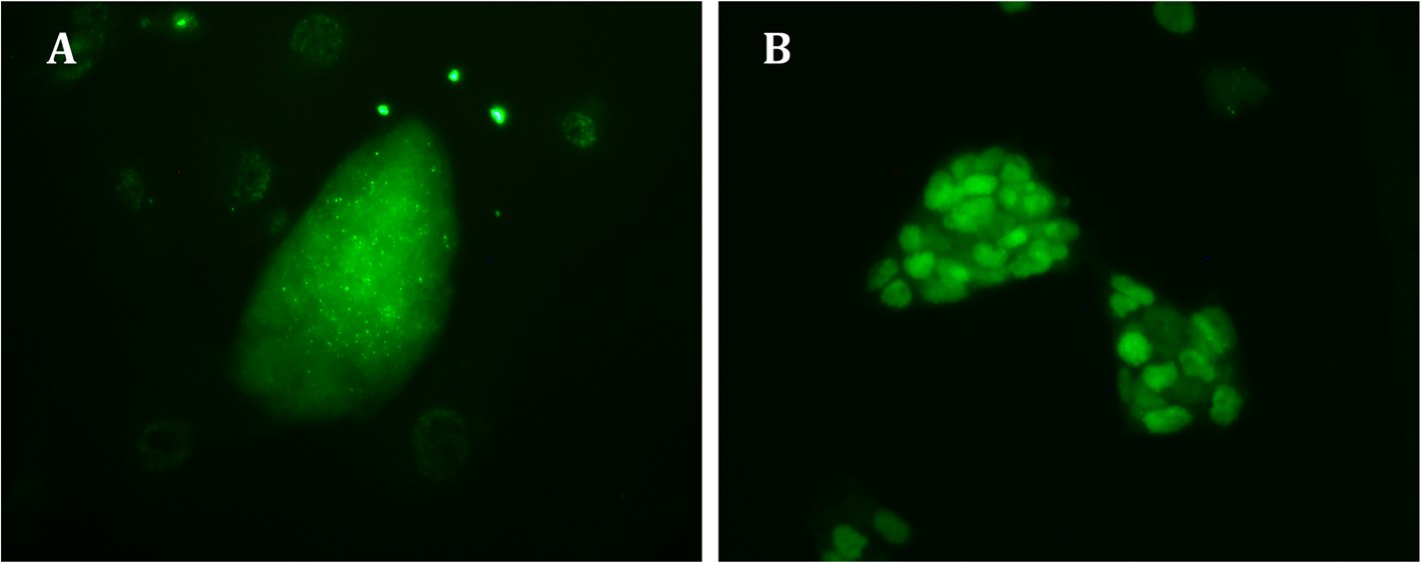
Immunofluorescence of NANOG in iPS colonies derived from bovine (**a**) and mouse (**b**) cells. × 200

**Fig. 6:**
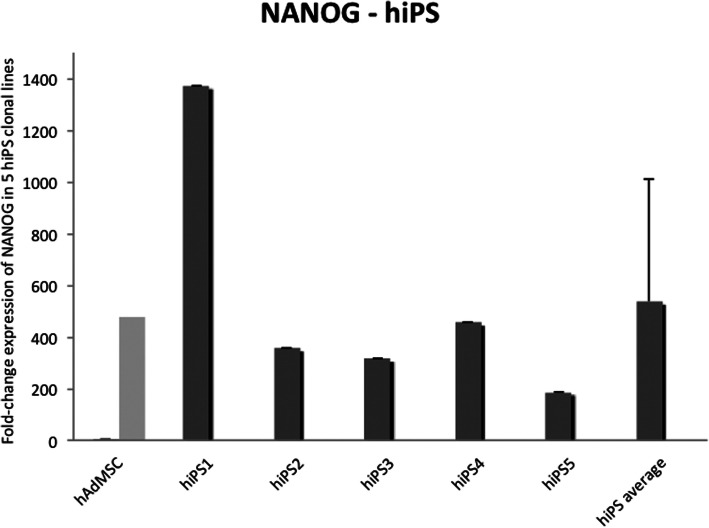
Detection of NANOG expression in hiPS colonies

Alkaline phosphatase enzyme (AP) and OCT4 were detected in iPSC colonies from all four species (Fig. 4). NANOG expression was detected in biPS and miPS by immunofluorescence (Fig. 5) and in hiPS clonal lines by RT-qPCR (Fig. 6). eiPSCs were further characterized elsewhere (Pessôa et al. 2019).

**Fig. 7:**
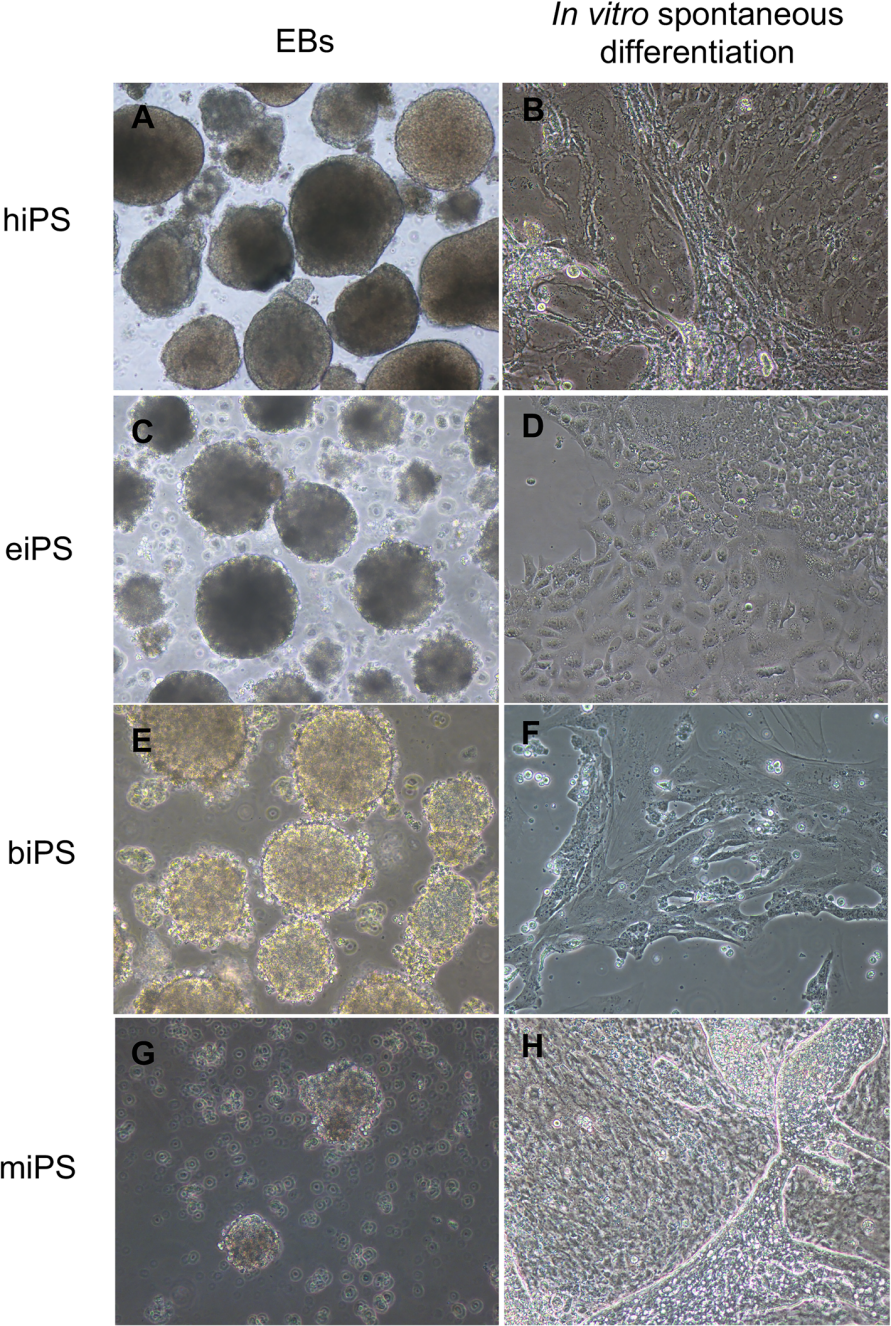
Embryoid bodies (**a**, **c**, **e**, **g**, × 100) and spontaneous in vitro differentiation (**b**, **d**, **f**, **h**; × 200) of hiPS, eiPS, biPS, and miPS

**Fig. 8:**
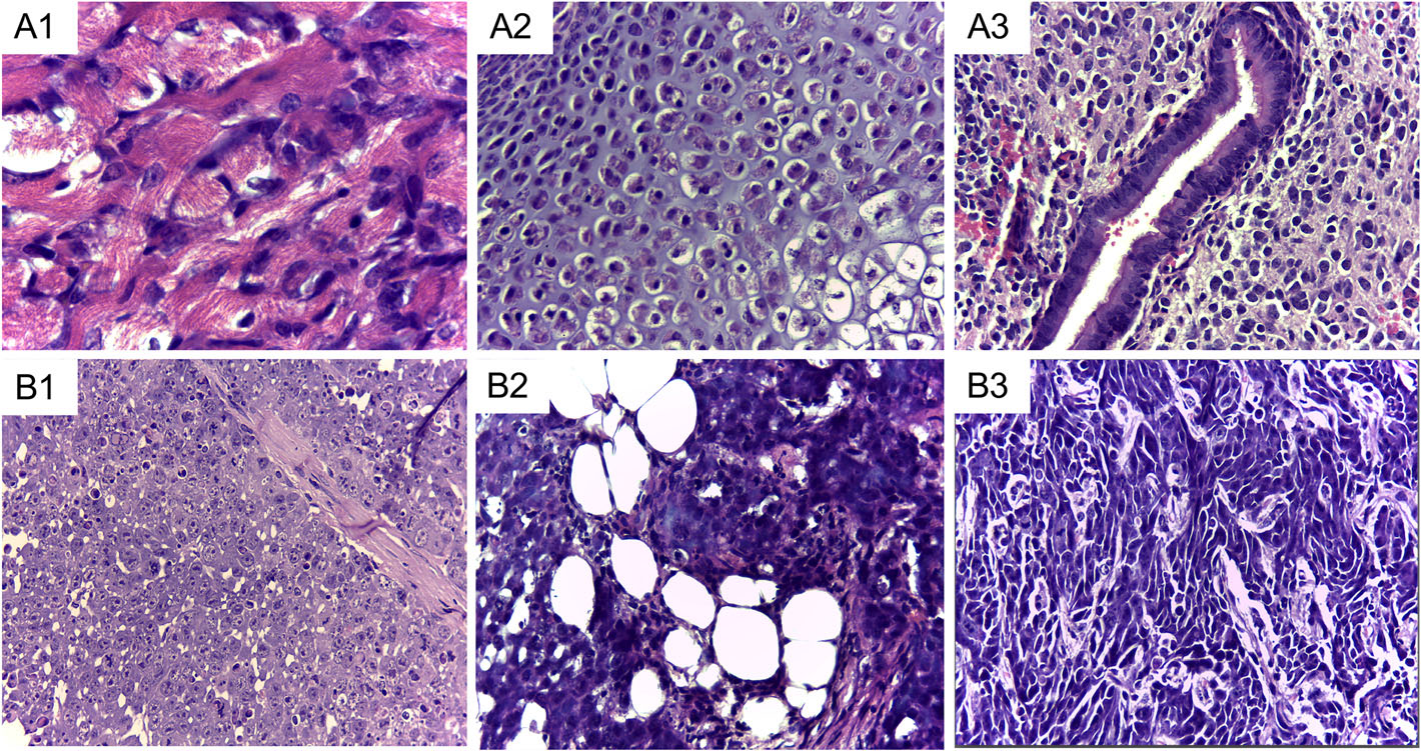
Photomicrograph of a teratoma derived from miPS showing three different tissues: (A1) striated muscle, (A2) cartilage, and (A3) pseudostratified ciliated epithelium. Photomicrograph of a tumor derived from biPS composed of (B1) undifferentiated tissue with high mitotic activity, (B2) adipose tissue, and (B3) fibroblast-like fusiform cells. Hematoxylin and eosin, objective × 40

**Fig. 9:**
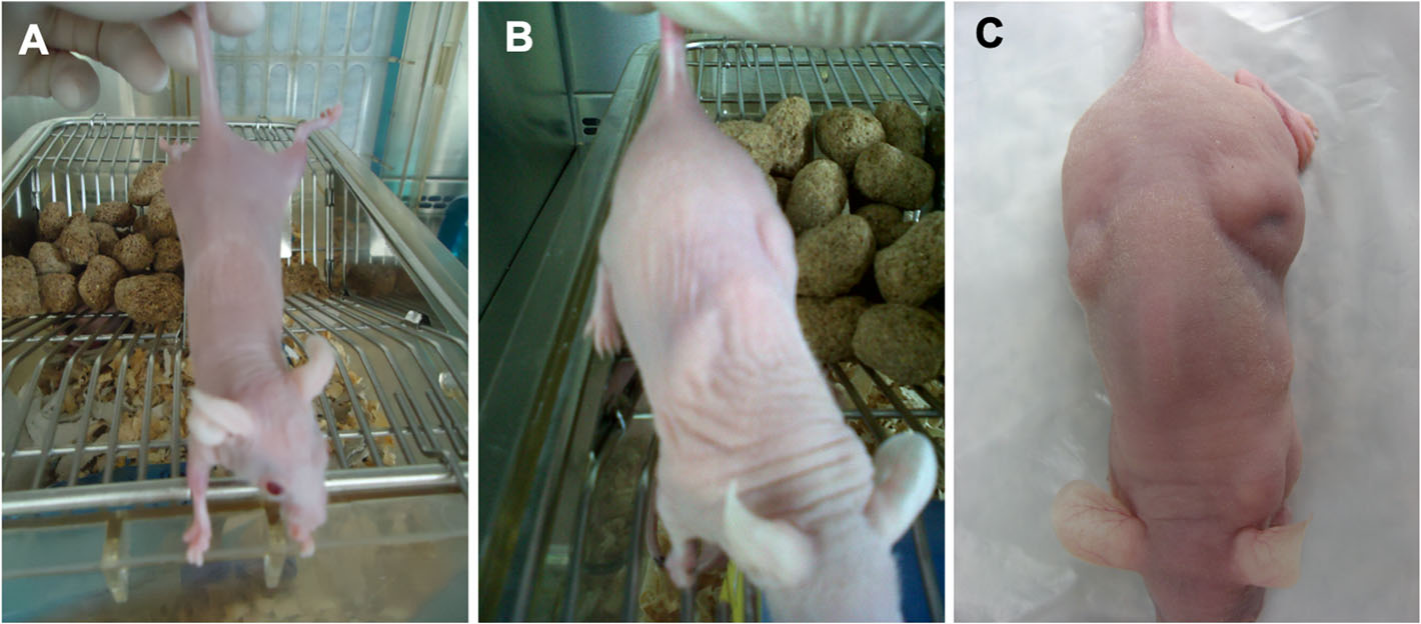
Balb/c nude mice injected with bFF (**a**); biPS, 30 days (**b**); and biPS, 37 days (**c**). The same animal is presented in **b** and **c**

Embryoid bodies are presented in Fig. 7. No teratoma formation was observed when human or equine iPS cells were inoculated in nude mice after 6 months. Tumors were collected from animals injected with biPSCs or miPSCs (Fig. 8) after approximately 2 months. Histopathological evaluation revealed the growth of at least 3 different tissues per lineage, including well- and poorly differentiated specimens. Animals that received mesenchymal cells or fibroblasts did not present teratoma formation. The macroscopic identification of teratomas derived from biPSCs in different periods is presented in Fig. 9.

## Discussion

Currently, the most common criteria for the characterization of human and murine embryonic stem cells include the presence of a typical phenotype, which is described by an increased nucleus/cytoplasm ratio; the expression of pluripotency markers; high telomerase activity; and in vitro and in vivo pluripotency [36]. In other species, however, these criteria were not yet reproducible until the advent of iPSC technology. In cattle, for example, isolation of embryonic cells results in colonies that may correspond to some of the criteria required for human and murine cells; however, in vitro culture of these stem cells revealed differences from the characteristics outlined above in several reports [37–41].

Possible causes for these inter-species differences are the difficulties of maintaining pluripotency conditions in vitro, as well as the lack of knowledge about factors that regulate stem cells isolated from embryos of domestic species. Therefore, in these species, they are called *stem-cell-like* [42].

Some studies have already reported bovine and equine potential iPSC cell lines with interspecific vectors (human, swine, or murine cDNAs), but obtained cells with divergent characteristics [8, 11, 43–45]. Huang et al. [46] reported the use of a non-integrative polycistronic vector containing bovine OCT4, SOX2, KLF4, and c-MYC driven by independent promoters. Media were supplemented with LIF, MEK1/2, and GSK3 inhibitors (2i); however, only quiescent non-proliferating cells were generated. Interestingly, this “iPS-like” state described by Huang and collaborators showed similarity to our non-proliferating bovine colonies obtained when human OKSM was used. Zhao et al. [47] reported the maintenance of biPSCs for more than 50 passages when reprogramming was achieved through piggyBac transposon integration of CAG-promoting OSKM, and Han et al. [10] were able to maintain biPSC cell lines for at least 16 passages when embryonic fibroblasts were used for reprogramming through the retroviral mechanism. Nonetheless, bovine reprogramming factors were also used elsewhere [47, 48], and the biPSCs presented showed characteristic pluripotency features, such as teratoma formation and pluripotency markers OCT4, SOX2, NANOG, and others (for extensive review, see Pessôa et al. 2019b).

eiPSCs have already been obtained by transposon-mediated or retroviral expression of human or murine OSKM or OSK (without c-Myc) in fetal or adult fibroblasts [49–51] [45, 52, 53], keratinocytes [54], blood and muscle cells [55], adipose tissue cells [45, 56], and umbilical cord tissue cells [45]. Overall, these cells presented different pluripotency features, such as teratoma and embryoid body formation, alongside the detection of pluripotency markers OCT4 and NANOG, as well as others, as recently reviewed [2]. Regarding medium supplementation, although there are reports of eiPS dependent of the combination of LIF and FGF [49–51, 53], and even only LIF [52, 54, 56], eiPSCs produced here were dependent exclusively of bFGF, as also seen in other reports [45, 55].

Herein, cellular reprogramming was performed in bovine and equine somatic cell cultures through the lentiviral transduction of interspecific OSKM (murine and human). We also generated murine and human iPSC cells, aiming to define a reproducible and controlled pluripotency induction protocol for domestic species. The iPS cells derived herein were positive for NANOG by immunofluorescence, which was used as a pluripotency reporter because no exogenous NANOG was used for reprogramming. The iPSC lineages were characterized after 10 passages and were maintained at least 30 passages in vitro. Exogenous expression was not silenced even after 30 passages, as expected and reported previously in other studies using viral vectors [57, 58]. Similarities and differences between species are summarized in Table 1.

**Table 1:** Summary of pluripotency-related characteristics of hiPS, eiPS, and biPS derived from fibroblasts or mesenchymal cells and miPS derived from fibroblasts

Exogenous transcription factors	hiPS	eiPS		miPS	biPS	
	human OSKM	Murine OSKM	Human OSKM	Murine OSKM	Murine OSKM	Human OSKM
Morphology	Flat	n.a.*	Flat	Dome-shaped	Dome-shaped	Dome-shaped
1st appearance of typical colonies	5 days	n.a.*	3 days	3 days	7 days	> 30 days
Replating	Manual	n.a.*	TrypLE	TrypLE	TrypLE	n.a.**
AP	+	n.a.*	+	+	+	–
EB	+	n.a.*	+	+	+	n.a.**
Tumor formation	–	n.a.*	–	+	+	n.a.**
LIF dependence for iPSCs generation	–	n.a.*	–	+	–	n.a.**

n.a.* not assessed due to no colony formation, n.a.** not assessed due to quiescent-like colonies

In this study, interspecific transcription factors were able to reprogram somatic cells into a pluripotent state. In fact, evidence that regulatory interactions are conserved among organisms has already been reported [59, 60]. When OCT4, SOX2, c-Myc, and KLF4 cDNA and protein are compared between species, both equine and bovine OSKM transcription factors are more similar to human than murine homologs [Table 2, mRNA similarity % (protein similarity %)]*. The reason why bovine fibroblasts were successfully reprogrammed with murine but not human OSKM is still unclear. However, our results stress the need for further research into a possible new mechanism of transcriptional networks.

**Table 2:** Homology of mRNA and protein of transcription factors OCT4, SOX2, c-Myc, and KLF4 between species

	*Equus caballus*				*Bos taurus*			
	OCT4	SOX2	c-MYC	KLF4	OCT4	SOX2	c-MYC	KLF4
*Homo sapiens*	92% (96%)	92% (91%)	88% (92%)	90% (92%)	90% (94%)	95% (99%)	86% (92%)	91% (94%)
*Mus musculus*	85% (88%)	88% (88%)	83% (90%)	87% (91%)	83% (88%)	88% (98%)	85% (90%)	88% (92%)

mRNA and protein (ncbi.nlm.nih.gov), respectively:

*Equus caballus* OCT4: XM_001490108.4, XP_001490158.1; SOX2: XM_003363345.2, XP_003363393.1; c-Myc: XM_001497991.3, XP_001498041.2; KLF4: XM_005605684.1, XP_005605741.1

*Bos taurus* OCT4: NM_174580.2, NP_777005.1, SOX2: NM_001105463.2, NP_001098933.1, c-Myc: NM_001046074.2, NP_001039539.1; KLF4: NM_001105385.1, NP_001098855.1

*Homo sapiens* OCT4: NM_001173531.2, NP_001167002.1; SOX2: NM_003106.3, NP_003097.1, c-Myc: NM_002467.4, NP_002458.2; KLF4: NM_004235.4, NP_004226.3

*Mus musculus* OCT4: NM_001252452.1, NP_001239381.1; SOX2: NM_011443.3, NP_035573.3; c-Myc: NM_001177353.1, NP_001170824.1; KLF4: NM_010637.3, NP_034767.2

## Conclusions

Herein, we showed the generation of equine and bovine stem cells in vitro through the expression of exogenous and interspecific transcription factors. Although a similar protocol was able to reprogram these cells, it was observed that they differ regarding requirements and characterization. The acquaintance and maintenance of pluripotency in vitro provide a powerful tool to improve the understanding of early development in these species and may also facilitate the production of genetically improved organisms if combined with other reproductive technologies.

## Data Availability

Not applicable.

## Abbreviations

iPSCs: Induced pluripotent stem cells
OSKM: Oct4, Sox2, KFL4, and c-Myc
biPSCs: Bovine induced pluripotent stem cells
miPSCs: Mouse induced pluripotent stem cells
eiPSCs: Equine induced pluripotent stem cells
hiPSCs: Human induced pluripotent stem cells
ES cells: Embryonic stem cells
AdMsc: Adipose tissue-derived mesenchymal cells
bFF: Bovine fetal fibroblasts
mFF: Murine fetal fibroblasts
eAF: Equine adult fibroblasts
hAF: Human adult fibroblasts
bADMscs: Bovine adipose tissue-derived mesenchymal cells
hADMSC: Human adipose tissue-derived mesenchymal cells
eADMSCs: Equine tissue-derived mesenchymal cells
IMDM: Iscove’s Modified Dulbecco’s Media
STEMCCA: Stem cell cassette
MEFs: Mitotically inactivated mouse embryonic fibroblasts
DMEM/F12 KO: Dulbecco’s modified Eagle’s medium/Ham’s nutrient mixture F12 knockout
KSR: Knockout serum replacement
bFGF: Basic fibroblast growth factor
pen/strep: Penicillin and streptomycin
LIF: Leukemia inhibitor factor
hLIF: Human leukemia inhibitor factor
mLIF: Mouse inhibitor factor
2i: iGSK3+iMEK
iGSK3: Glycogen synthase kinase 3 inhibitor
iMEK: Mitogen-activated protein kinase inhibitor
AP: Alkaline phosphatase
PFA: Paraformaldehyde
PBS: Phosphate-buffered saline buffer
BSA: Bovine serum albumin
RT-qPCR: Reverse transcription quantitative polymerase chain reaction (real-time PCR)
EBs: Embryoid bodies
H&E: Hematoxylin and eosin stain
